# Physical Comorbidities and Their Relationship with Cancer Treatment and Its Outcomes in Older Adult Populations: Systematic Review

**DOI:** 10.2196/26425

**Published:** 2021-10-13

**Authors:** Mathew George, Alexandra Smith, Sabe Sabesan, Geetha Ranmuthugala

**Affiliations:** 1 North West Cancer Centre Tamworth Hospital Hunter New England Local Health District Tamworth Australia; 2 School of Rural Medicine University of New England Armidale Australia; 3 Townsville Cancer Centre Townsville University Hospital Townsville Australia

**Keywords:** comorbidities, cancer, chemotherapy, geriatric, quality of life, morbidity, treatment, older adults, review

## Abstract

**Background:**

Cancer is one of the predominant causes of morbidity and mortality in older adult populations worldwide. Among a range of barriers, comorbidity particularly poses a clinical challenge in cancer diagnosis, prognosis, and treatment owing to its heterogeneous nature. While accurate comorbidity assessments and appropriate treatment administration can result in better patient outcomes, evidence related to older adult cancer populations is limited as these individuals are often excluded from regular clinical trials due to age and comorbid conditions.

**Objective:**

To determine the prevalence of physical comorbidity and the impact of physical comorbidities and rurality on treatment and its outcomes in older adult cancer populations.

**Methods:**

Scientific databases Embase and PubMed were searched for published scientific literature on physical comorbidity and older adult cancer patients. Google Scholar was searched for scholarly literature published in nonindexed journals. Snowballing was utilized to identify research papers missed in the above searches. Included studies : (1) reported on original research involving cancer patients; (2) included patients aged 65 years or older; (3) had patients receiving cancer-related treatment and (4) cancer survivors; (5) reported on physical comorbidity as a variable; (6) were published in English; and (7) conducted from any geographical location.

**Results:**

In total, 29 studies were selected for data extraction, evidence synthesis, and quality assessment. In these, comorbidities ranged from 37.9%-74.3% in colorectal cancer, 74%-81% in head and neck cancer, and 12.6%-49% in breast cancer. Moderate comorbidities ranged from 13%-72.9%, and severe comorbidities from 2.5%-68.2%. Comorbidity increased with age, with comorbidity affecting both treatment choice and process. Physical comorbidities significantly affected treatment initiation, causing delay, toxicity, and discontinuation. Older adult cancer patients were given less vigorous and nonstandard treatments and were also less likely to be offered treatment. Where patients are given more vigorous treatment, several studies showed better survival outcomes. Appropriate treatment in older adult cancer patients increased both overall and disease-related survival rates. None of the studies noted rurality as a distinct variable.

**Conclusions:**

This systematic review concludes that there is evidence to substantiate the adverse effect of comorbidity on treatment and survival outcomes. However, the mechanism by which comorbidity impedes or impacts treatment is unknown in many cases. Some low-quality evidence is available for considering the functional status and biological age in treatment decisions. Future studies that substantiate the value of comprehensive older adult assessments before treatment initiation in cancer patients, including assessing the nature and severity of comorbidities, and additional consideration of rurality as a factor, could lessen the effect of comorbidities on the treatment process.

## Introduction

Cancer is one of the predominant causes of morbidity and mortality in older adult populations worldwide, particularly in developed countries owing to the proportionately high aging population [1-5]. Frailty, comorbidities, financial burden, treatment-related adverse effects, and lack of social support, transportation, and treatment facilities are some of the hindrances in cancer treatment among older adult populations [6-9]. Of these factors, comorbidity poses a major clinical challenge in cancer diagnosis, prognosis, and treatment owing to its heterogeneous nature in terms of number as well as severity [5,10-13].

Accurate comorbidity assessments and appropriate treatment administration can result in better treatment outcomes in older cancer patients [10-14]. However, evidence related to the impact of comorbidities and their relationship with treatment outcomes in older adult cancer populations is limited as these individuals are often excluded from regular clinical trials due to age and comorbid conditions [15,16]. Recently, there has been an increased interest among researchers to specifically study the treatment of and outcomes in older adult cancer populations. This review focuses on older adult cancer patients and aims to examine the prevalence of comorbidity among the older adult cancer population and to understand the impact of physical comorbidities on (1) treatment (delay in treatment initiation, completion, dose alteration, or treatment-related adverse effects) and (2) outcomes (survival and quality of life [QoL]) in the population.

## Methods

### Reporting Guidelines Used

This review was undertaken using established criteria for the conduct and reporting of systematic reviews given by the 2009 PRISMA (preferred reporting items for systematic reviews and meta-analysis) [17], including those identified by Moher et al [18].

### Search Strategy

**Figure 1:**
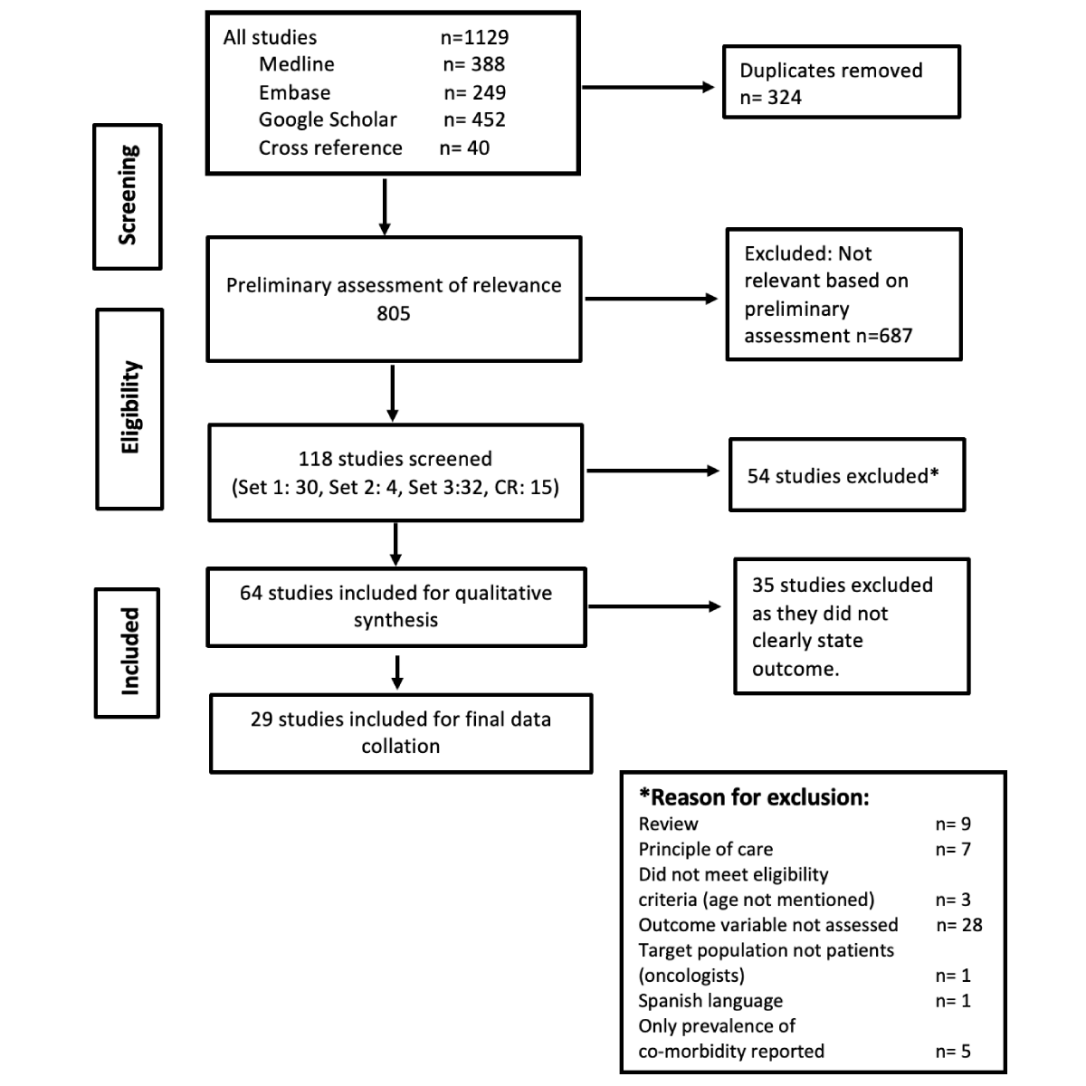
PRISMA flowchart of search results and study selection. PRISMA: preferred reporting items for systematic reviews and meta-analysis.

Embase and PubMed were searched for peer-reviewed literature published between January 1, 1991, and June 2019. Google Scholar was also searched to identify scholarly publications not identified from the database searches. Searches were undertaken using a combination of medical subject heading terms, Emtree indexed search terms, and specified keywords relating to the target population and subject matter, including “geriatric cancer,” “cancer treatment,” “physical comorbidity,” “survival,” “quality of life,” and “treatment outcomes.” The search strategy and terms used to search the Embase, PubMed, and Google Scholar databases are reported in Multimedia Appendix 1. In addition, snowballing was undertaken to identify scientific literature cited within papers that may have been otherwise missed from the above searches. The searches were limited to literature published in English. Search results were downloaded to Covidence [19] to assist with the review and data extraction. The process and results of the search are presented in the PRISMA flow chart (Figure 1).

Of note, initial searches and subsequent browsing were undertaken for articles within the above parameters that also included “rurality” or related terms in their description of study design, with specific reference to variables for analysis. This process yielded no results, and therefore, the overall scope of the systematic review was necessarily narrowed. However, as discussed later in this review, the absence of literature in this respect highlights a significant gap for further research development. The present systematic review also did not include randomized controlled trials, as the review aimed to understand the impact of comorbidities on treatment outcomes. In addition, this approach reflects the approach and findings of an existing systematic review in the broader field [20].

### Selection of Studies

Two independent reviewers, MG and AS, initially reviewed a number of the articles by title and abstract, using specific eligibility criteria mentioned as follows in order to assess the level of agreement. Once this agreement and consistency of eligibility criteria application were reached via agreement on at least 5 of 6 criteria for articles in reviewers’ initial screening selections, the reviewers continued to screen the remaining articles for relevance against the criteria. As previously mentioned, only literature published in English was included in this stage.

Eligibility criteria for study inclusion included: (1) reported on original research involving cancer patients; (2) included patients aged 65 years or older; (3) patients were receiving cancer-related treatment; (4) those who are survivors; (5) reported on physical comorbidity as a variable; and (6) were published in English. Research from any geographical location (ie, urban or rural) was included.

Population-based studies that included a subgroup analysis of older adults (ie, 65 years and older) were also included in the present systematic review. This assisted in accounting for the results of participants of younger ages.

The study was chosen for the review if both the reviewers individually approved it, and, in cases of uncertainty, the article was included for full-text screening. Each reviewer then screened the full text of selected studies individually to ensure that the articles met all inclusion criteria. In cases of any discrepancy, consensus was reached after meticulous discussion by the reviewers.

### Data Extraction

After completion of screening, data from included articles were extracted manually by the two reviewers. The reviewers then independently assessed and scored the individual studies using the National Institute of Health quality assessment tool for observational cohort and cross-sectional studies [21]. The tool consists of 14 questions relating to the risk of bias and other indicators of quality. The average scores of the reviewers across these indicators were then calculated to categorize the studies as “high,” “moderate,” or “low” quality.

## Results

In total, 1129 studies were identified from the electronic database searches, and 40 studies were obtained through cross-reference. This was reduced to 805 studies after removing 364 duplicates and reduced further to 118 studies based on the process of title and abstract screening. After excluding 364 duplicates and 686 articles that did not meet eligibility criteria, articles were then identified and agreed upon as potentially relevant.

A total of 118 papers remained following this screening process, with the exclusion of a further 54 papers as per the exclusion criteria. The process and outcomes are illustrated in Figure 1 (PRISMA flowchart). Excluded were book reviews (n=9); studies that did not report the age of participants (n=3); target study population was not comprised of patients but rather was comprised of a general population potentially including both patients and nonpatients (n=1); outcome variable was not assessed (n=28); and only the prevalence of comorbidity, rather than type or other details, was reported (n=5). Studies were also excluded if they did not indicate the principle of care (ie, treatment regimen and treatment modality; n=7). No editorial reports were obtained during the initial search and therefore did not account for any excluded articles. Case reports were excluded at the preliminary assessment of relevance stage, in which 687 articles in total were excluded. Figure 1 illustrates the number of studies identified and included and the reasons for exclusion.

Based on the inclusion and exclusion criteria, a total of 29 studies were selected for data extraction and evidence synthesis and then assessed for quality.

### Quality Assessment

Quality assessment of the studies revealed 1 study to be high quality [22], 5 studies of moderate quality [23-27], and 23 studies [28-50] to be of low quality. Those of low quality were those studies in which: sample size justification, power description, or variance and effect estimates were not provided or were lacking; exposures of interest were measured prior to the outcomes being measured; and there were high rates of attrition owing to loss to follow-up after baseline (while this was not mentioned by all studies, approximately 13 studies noted a <20% attrition rate). We did not exclude any study from the final review based on this quality assessment, and its results are presented in Multimedia Appendix 2. The following sections discuss principal findings consolidated from all 29 studies, focusing on identifying research gaps for further elaboration.

### Study Characteristics: Summary

A summary of the studies included is reported in Multimedia Appendix 3, and the characteristics and quality of each study are provided in Multimedia Appendix 2. Next, we elaborate on the study characteristics summary, with cited studies specified in the following results sections.

All studies were observational in nature, comprising cross-sectional, prospective, or retrospective studies. Most studies were retrospective (n=27) in nature. Fifteen of the 29 studies obtained their data from data registry reviews, and the remainder were based on data obtained from patient hospital records.

Sample size ranged from 59 in a small study from Portugal [24] to 61,740 in a retrospective study from the United States [33]. Big data drawn from database record reviews and patient hospital records are likely to include patients from various geographic settings. However, the difference between urban and rural settings and their impact on comorbidity were not specifically studied. Only 1 of the 29 studies included in this review examined this difference in comorbidities between urban and rural settings [45].

Studies on colorectal cancers (n=11) were the most common, followed by head and neck cancers (n=5) and breast cancers (n=4). All the studies focused on single-site cancers and none on metastatic cancers. The tumor stage was described in all but 4 of the studies, with a marginal focus on stage III cancers overall. The common covariates examined in the studies were age (100%), sex (56.7%), stage of cancer (50%), and ethnicity (30%).

Different tools of assessment were used in the studies to assess comorbidity. The Charlson Comorbidity Index (CCI), with or without modification, was the most commonly used tool (56.7%) in the studies [22,24,28,29,31,33,35,36,41,46-48,50]. Three studies used the Kaplan-Feinstein Index [3,32,39] and the adult comorbidity evaluation index [25,34]. One study assessed the QoL in participants using the European Organization for Research and Treatment of Cancer Quality of Life Questionnaire (EORTC) scale [32], and 1 study used the activities of daily living scale [44] to elucidate daily physical activity capabilities and limitations

Comorbidities were reported based on the severity as either mild, moderate, or severe (n=8) or based on a numerical scale ranging from 0 to ≥8 (n=16), while one study reported on both [46]. Four studies did not mention any categorization of comorbidities. Reported comorbidities were classified under cardiovascular, diabetes, gastrointestinal, pulmonary, renal, neuromuscular, hematopoietic, psychiatric problems, and others (eg, obesity, arthritis, HIV/AIDS, poor vision and hearing), with the specific type of comorbidity not mentioned in 6 studies. Diabetes was most commonly mentioned, being included in about 40% of the studies, followed by hypertension (36.7%) and cardiovascular-pulmonary and cerebrovascular problems (30%).

Chemotherapy was found to be the most commonly used treatment (75.9%), followed by surgery (51.7%) and radiotherapy (17.2%). These therapies were either used alone (n=18) or in combination (n=11).

### Comorbidities

#### Prevalence of Comorbidities

The prevalence of the presence of any comorbidities among patients with colorectal cancer ranged from 37.9% to 74.3%, 74% to 81% [33] among those with head and neck cancer, and 12.5% [38] to 49% [29] among those with breast cancer as reported in Multimedia Appendix 4. Moderate comorbidities were reported ranging from 13% [33] to 72.9% [24] and severe comorbidities ranged from 2.5% [49] to 68.2% [41] in the study population of selected studies. The proportion of patients classified under varying severity levels of comorbidity was not mentioned in 3 studies [34,43,44]. Patients with no comorbidities ranged from 0.7% to a maximum of 87.4% [28]. Klepin et al [23] reported the median total number of comorbidities as 2 (range 0-10) and the median comorbidity burden score as 3 (range 0-25) among patients. Tan et al [50] reported the median CCI as 3 (range 2-10) to indicate the severity of comorbidities. Koroukian et al [38] scored multimorbidities which included functional limitations and geriatric syndromes along with comorbidities. About 21.2% of patients had no multimorbidity, and 78.8% had scores of 1-3. Miguel et al [24] categorized 72.9% as fit and 27.1% as vulnerable categories. Sanoff et al [48] elaborately described the prevalence of comorbidities from the surveillance, epidemiology, and end results program, the New York State Cancer Registry, and the National Comprehensive Cancer Network databases individually.

#### Impact of Comorbidities on Cancer Treatment

Across the selected studies, comorbidities were identified as impacting cancer treatment in a number of ways; however, the causative mechanism of this impact and the degree of impact was neither consistently studied nor reported, making it challenging to draw overall conclusions. The impact of physical comorbidities on cancer treatment and salient findings of each study, as these were statistically analyzed and reported by study authors, is summarized in Tables S3 and S4 of Multimedia Appendix 4 . Major themes included impact of comorbidities on cancer treatment choice, initiation, dose reduction, and other alterations including delay, adverse effects, and discontinuation.

The choice of treatment was noted as affected in some way due to comorbidities in 19 studies [22,24,25,27,28,30-33, 36,38,40,42-44,46-48,50]. Nonstandard treatment and less aggressive treatment were given for older geriatric patients during both primary and secondary treatment regimens, the main factors cited in this being age and physical comorbidities. Hoeben et al [25] reported that the type of chemotherapy had to be modified in 3% out of 57% of patients who received chemotherapy.

Comorbidity affecting treatment initiation was reported in 3 studies [22,25,45]. Hu et al [45] revealed that patients aged 75-79 years were 71% less likely than those aged 65-69 years (OR [odds ratio] 0.29, 95% CI 0.25-0.34) to initiate chemotherapy, and patients with >2 on the comorbidity index were 63% less likely (OR 0.37, 95% CI 0.33-0.42) to initiate chemotherapy after surgery. Age, comorbidity, and marital status were significant predictors for chemotherapy initiation, which showed a model variance of 92.6% in the chi square test. Gross et al [22] studied the presence and absence of individual comorbidities and the initiation of adjuvant therapy. Initiation of therapy for patients with and without coronary heart failure was 36.2% vs 64.9% (OR 0.49, 95% CI 0.40-0.60), with and without chronic obstructive pulmonary disease (COPD) was 55.2% vs 61.5% (OR 0.83, 95% CI 0.70-0.99), and with and without diabetes was 58.3% vs 60.7% (OR 0.81, 95% CI 0.68-0.97). Hoeben et al [25] reported that chemotherapy was not initiated in 43% of patients due to age, comorbidity, or performance status, whereas patient preference accounted for only 17% of noninitiation decisions following surgery [25].

Dose alteration was identified and discussed in 7 studies [23-25,27,29,35,42]. An increase in comorbidities was related to dose modification in patients for ≥2 vs <2 comorbidities and was reported as 40% vs 31% (*P*<.05) by Goede et al [35] and 59% vs 46% (*P=*.03) by Klepin et al [23]. Dose reduction was also related to adverse effects from treatment (n=19, 9%) in patients [29]. Hoeben et al [25] reported that 18% and 28% of patients who received chemotherapy underwent alterations in dose and number of sessions, respectively, and in 3% of patients, dose reduction was made before the initiation of treatment. This dose reduction was noted as being not significantly related to age or comorbidity. Jørgensen et al [42] observed that dose reductions in the carboplatin and taxane treatment group in ovarian cancer patients were related to toxicity, but in 17%, it was due to comorbidity or age; however, no significant difference was found based on age for the group receiving only the carboplatin treatment regimen. In rectal cancer, 29.8% of patients had dose reductions (34.3% for 0-1 CCI and 16.7% for >2 CCI; *P=*.22) [24]. On the contrary, Grønberg et al [27] found no significant differences during therapy and posttherapy in patients (without drug modification) with severe comorbidity.

Treatment delay was examined in 3 studies [25,29,43]. Hoeben et al [25] reported that there was modification in time course between successive chemotherapy sessions in 23% of patients, but this was not related to age or comorbidity. Ferrero et al [43] reported no difference in delay between the age groups 70-75 years and >75 years or based on frailty, but this result was not significant. However, O’Connor et al [29] reported an unplanned delay in treatment for more than a week in about 20% of patients due to toxicity which was significantly related to a history of comorbidities, especially diabetes, hypertension, and low creatinine clearance. An anthracycline-based chemotherapy regimen, CCI ≥1, and hypertension were predictors for treatment delay. A CCI ≥1 was a significant predictor for delay in chemotherapy administration. Age was also a risk factor for delayed treatment.

Treatment discontinuation was reported in 9 studies [22,23,27,29,35,41-43,45]. The most common factors cited in treatment discontinuation were disease progression, toxicity, and patient preference [23,41]. Hu [45] reported that older patients (*P*<.05) and a <2 comorbidity score (OR 0.63, 95% CI 0.52-0.75) were significant predictors for early discontinuation, and age at diagnosis was the strongest predictor of treatment discontinuation. Similar results were reported by O’Connor et al [29] (OR 4.43, 95% CI 1.55-12.69; *P=*.045 for >75 years and <75 years). Gross et al [22] found no significant association between individual comorbid conditions and completion of treatment. According to Grønberg et al [27], 69% of patients completed chemotherapy (*P=*.08); however, the rate was lesser in patients with a severe comorbidity.

The overall response rate for treatment was also found to be lesser in patients with higher comorbidities (75% vs 85% for ≥2 vs <2 comorbidities, respectively; *P*<.05), but no significant variation was found when the results were adjusted for age and treatment, suggesting that patients with high comorbidity were biased to receiving less vigorous treatment [35]. Ferrero et al [43] reported that complete response to treatment was greater among the 70-75 years age group than among the <75 years age group (60% vs 28.9%, respectively; *P=*.005). Also, no significant difference between age groups was found for treatment discontinuation due to toxicity in ovarian cancer patients (*P=*.28) [43]. A similar result was found with respect to the carboplatin-only treatment regimen in a study done by Jørgensen et al [42], whereas in the carboplatin and taxane regimen, performance status and severity of comorbidity were predictors for treatment discontinuation.

Treatment toxicity, adverse effects, or postoperative complications were observed in 14 studies [22-25,27,29,33-35,37,40,41,43]. Goede et al [35] analyzed the individual comorbidities with treatment toxicity and reported no relationship between the variables. However, Grønberg et al [27] observed that the incidence of fever was high in patients with severe comorbidities and also identified that minor comorbidities were not registered in their study, which might have contributed to the result. This suggests the importance of recording the comorbidities, their types, occurrence, and nature in-depth without omitting any details in order to decrease treatment-related adverse effects. In lung cancer, the hematological and nonhematological toxicities were 3% and 24%, respectively [37]. Houterman et al [40] reported no significant difference between treatment complications and comorbidities, irrespective of age. Peters et al [34] reported on recipient site and medical complications out of which the latter was found to be significantly present in head and neck cancer patients with ≥2 comorbidities (OR 2.89, 95% CI 1.71-4.84; *P*<.001). Phaibulvatanapong et al [41] presented a detailed account of treatment-related complications with adverse effects (grade 3-5) in 83.4% and severe toxicity in 42.4% of patients, both of which were related to performance status in a mixed cancer study population (*P*<.05). Ferrero et al [43] reported a higher rate of postoperative complications in high-frailty patients compared with low-frailty patients (23.5% vs 4.3%; *P*=.03). Tan et al [50] similarly reported worse postoperative complications in patients with a CCI >3 or those who had emergency surgery. The study also reported worse perioperative complications and higher death rates among those >85 years old. Hospitalization was not related to congestive heart failure (CHF), COPD, or diabetes, irrespective of whether individuals received treatment [22]. Conversely, Genther and Gourin [33] reported that comorbidities were related to emergency hospital admission (relative risk1.21, 95% CI 1.06-1.38; *P=*.005) but not to postoperative complications.

Treatment-related toxicity (25.4%, 52%, and 9%) [24] was also another reason cited for treatment discontinuation (1.7%, 15%, and 20%) [23] and dose reduction (29.8%, 51%, and 9%)[29]. Adverse effects varied with the type of treatment (52% vs 41% for those receiving vs not receiving adjuvant chemotherapy, respectively) [25]. O’Connor et al [29] found that history of hypertension is a predictor for poor tolerance of chemotherapy causing treatment delay (OR 2.51, 95% CI 1.02-6.20; *P=*.046).

Some of the selected studies have noted patients’ personal preference in treatment choice and discontinuation [24,26,32,41,42,50]. For example, Derks et al [32] reported that about 18% of the patients above 80 years of age refused to undergo treatment. Patients diagnosed in more recent years (ie, 2009 or later) were more likely to receive and complete treatment [45,47]. These studies overall show that increased age correlates with an increased likelihood of a patient declining treatment; however, the studies do not identify the specific reasons for this (eg, the impact of comorbidity, impact of function or nonfunction, and so on).

Therefore, several of the selected studies show a strong association between comorbidities and treatment dose alteration, noninitiation of treatment, treatment choice, and early discontinuation of treatment. Due to significant variation in cancer types or sites, patient cohorts, recording of comorbidities, and several other variables, it is, however, difficult to draw clear conclusions regarding the influence of comorbidities in particular on the treatment decisions and the effects among the broader patient population and that of older cancer patients in particular.

### Quality of Life and Survival Related Outcomes

Two studies documented health-related QoL of older adult cancer patients [27,41], while 23 studies reported overall progression-free and disease-free survival, and 4 studies did not include a QoL or survival component [31,33,36,45]. Hospital readmission (n=3) [22,29,37] was also investigated in several studies. Of note, while the inclusion criteria included both patients receiving treatment and patients who were survivors of cancer as separate cohorts, all studies reported both on patients currently receiving treatment and who had completed treatment, and none were specific to survivors as a singular cohort only. The following summarizes findings from these 23 studies, with a specific focus on their reporting of survival, comorbidity, age, and treatment relationships.

Comorbidity, especially development of multimorbidities, is a strong prognostic factor for survival in cancer patients. Comorbidity was an independent factor in determining specific and overall survival (OS) [35]. 30-day mortality was greater in individuals aged over 80 years than in those aged 60-79 years (12% vs 3%, respectively; *P*=.02), and OS was greater in the latter group (30.1% vs 50.5%, *P*<.001) [49]. Berglund et al [28] reported that higher cancer-related and noncancer-related mortality was seen in patients with severe comorbidity both in early and advanced stages of cancer. Also, the hazard ratio (HR) was significantly higher with severe comorbidity in early breast cancer patients during the follow-up.

Moderate comorbidity increased the risk of mortality twice compared to those without comorbidity, even after adjusting for age, functional status, and treatment (adjusted odds ratio [AOR] 1.98, 95% CI 1.37-2.85; *P<*.001 [51]; HR 1.71 95% CI 1.15-2.56; *P=*.007) [26]. It was observed that older patients with pre-existing comorbidities were less likely to be suggested for both primary and secondary treatment (AOR >75 years 8.7, 95% CI 2.3-32.4; AOR <75 years 1.2, 95% CI 0.3-4.5 [46]; 25% vs 38%, respectively [40]; OR 0.63, 95% CI 0.58-0.69) [28]. Age and comorbidity were also independently related to reduced chances of being offered treatment [46]. Houterman et al [40] reported that in patients <70 years, moderate (HR 2.43, 95% CI 1.27-4.66) and severe (HR 2.87, 95% CI 1.40-5.90) comorbidities significantly increased the risk of mortality, while in patients ≥70 years, severe comorbidity (HR 2.97, 95% CI 1.12-7.86) significantly increased the risk of mortality. Treatment was not a significant prognostic factor when the age and severity of comorbidity were adjusted [40]. However, studies have proved that providing treatment or completing the treatment schedule reduces the rate of mortality irrespective of comorbidity (adjusted hazard ratio 1.43, 95% CI 0.57-3.60 [46]; HR 0.70, 95% CI 0.64-0.76 [22]; crude 5-year survival: 51% vs 32%; HR 0.5; *P*<.001 [47]; HR 0.5 [35]; 52% vs 34% *P*<.001; HR 0.73, 95% CI 0.55-0.98) [25]; 92% vs 66%; *P=*.013 [29]).

Falch et al [49] identified that with increased age, there was an increase in complications postsurgery, which led to higher mortality rates (≥80 years vs 60-79 years: 35% vs 17%, respectively; *P=*.009). CHR (HR 1.83, 95% CI 1.14-2.93) and noncerebrovascular neurological conditions (HR 1.96, 95% CI 1.12-3.42) influenced the survival rates of colon cancer patients [46]. One important finding by Koroukian et al [44] and Koroukian et al. [30] is that the association between survival and comorbidity may not be significant in the absence of functional limitations and geriatric syndrome. Poor physical functioning in QoL assessment was observed in the presence of high comorbidity [27], and the performance status of an individual is also a strong predictor for survival [26]. Derks et al. [32] observed poor QoL in patients who did not receive standard treatment, while the prognostic value of comorbidity was retained even after adjusting other variables [35,40].

In line with the above findings, Ferrero et al [43] reported better survival in less-frail patients (56 vs 27 months). There was a trend for a better OS in the low-frailty cohort (median 56 vs 27 months; *P*=.07). Ferrero et al [43] reported that high-frailty patients had poorer performance status (*P<*.001) and a higher incidence of hypertension (*P*=.001), diabetes (*P*=.001), obesity (*P*=.01), and chronic renal failure (*P*=.05) when compared with low-frailty patients. Miguel et al [24] also reported comorbidity as an independent predictor of OS. They also reported no difference in mean disease-free survival, grade 3 to 4 toxicity, and dose reduction between the groups.

## Discussion

### Principal Findings

The reviewed studies confirmed the association of physical comorbidities and treatment in older adult cancer patients. However, the strength of evidence is lesser as a majority of the studies were of low quality. The studies included in this systematic review had heterogeneous study designs, cancer populations, study settings, measurement scales, and reporting parameters of comorbidities, thus not permitting data pooling for a meta-analysis. Nonetheless, the results obtained do highlight several gaps and factors that, if further investigated and addressed, may contribute to a better understanding of the potential effects of different treatment and management approaches for cancer in older adult patients with comorbidities. In addition to the existing evidence, the review pointed towards clear gaps in research and clinical service provision in this field. Research priorities need to be clearly stated by international agencies to establish the prevalence, patterns, impact, and treatment of comorbidities in older adult cancer patients. There is a need to explore the difference in care patterns of cancer patients in urban and rural settings. Similarly, more evidence from low-income countries needs to be synthesized to investigate the relationship between comorbidity and treatment in cancer patients in those settings.

Regardless, as per the American Society of Clinical Oncology (ASCO) guidelines, older adults are to undergo a comprehensive geriatric assessment (GA) before deciding on their cancer treatment to identify the best option for them. By doing so, vulnerabilities among those aged 65 years and above can be detected because it is recommended that the GA is used as intended to guide treatment decisions in the cohort comprised of older patients with cancer [52].

Many of the selected studies have also supported the association of increased age with increased comorbidity. Studies clearly confirmed that age influences the treatment process and treatment method among older patients. Among patients, higher comorbidity was observed with increasing age [44,49], and an increase in the pace of disease progression in older patients was further observed despite the comorbidity burden being corrected [28]. Age at diagnosis was the strongest predictor for completion of treatment in older adult cancer patients [26]. It was also an independent predictor for the type and aggressiveness of treatment received and discontinuation of treatment [31,32]. The effect of age was observed even after adjusting for the comorbidity factor [34]. It has been observed that less vigorous and nonstandard treatment regimens were suggested to patients based on increasing age, even in cases where the patient may be capable of withstanding more aggressive treatment [33]. Jørgensen et al [42] found that a subgroup of undertreated patients with less aggressive treatment would have been able to endure standard treatment. The outcomes of adjuvant treatment were not affected by advancement in age in the study conducted by Sartafi et al [46]. Hence, studies have recommended considering biological age and functional status for treatment choice and not merely chronological age [29,31,40].

Studies assessed show that comorbidity is a direct confounder rendering competing risks for morbidity and mortality. Higher comorbidity diminished functional status [29], increased the rate of hospitalization [48], resulted in dose modification [44], and is an independent predictor for in-hospital death [25]. Functional limitation and “older adult syndrome” are also related to not receiving treatment [28,31]. Severity of comorbidity was a predictor for patients not receiving standard treatments in the ≥70 years age group (*P*<.05) [26]. Sarfati et al [46] reported that 32 out of 51 patients (63%) of >75 years of age (AOR 8.7, 95% CI 2.3-32.4) and 13 out of 16 patients (81%) with a comorbidity score >3 (AOR 20.1, 95% CI 4.2-95.6) were not offered chemotherapy. With increasing comorbidity, the treatment offered to patients was less vigorous [30,35,42], with age and comorbidity independently affecting the chances of receiving treatment [36]. Comorbidity also affected the disease prognosis negatively [30,31,38,43]. Adjuvant therapy yielded better outcomes in patients who did not suffer from CHF, COPD, or diabetes mellitus, thus showing the association of comorbidity with treatment response [48]. Hypertension also resulted in treatment delay and resulted in greater rates of hospitalization [32]. The effect of comorbidity on survival persisted after adjustment for other variables like age, gender, and cancer site, although combinations of therapies were seen to improve outcomes in patients with high comorbidity [43].

Both age and comorbidity are related to treatment response [29]. In the context of cancer, assessment of comorbidities is an appraisal of the effect of cancer and its treatment on the physical, mental, and social health of patients. Therefore, the use of comprehensive older adult assessments in cancer patients during treatment decisions should be strengthened [31]. Although the CCI is a widely used tool, Phaibulvatanapong et al [41] reported that it would not be suitable for comorbidity assessment, specifically for cancer patients, as cancer is one of the scoring components of CCI and might show an unjustified high score for metastatic patients. As such, significant consideration must be given to the consistent administration of the comprehensive geriatric assessment as per the ASCO guideline for geriatric oncology [52].

### Limitations

In this systematic review, contradictory findings on age and survival were reported. OS was significantly better in patients aged less than 75 years (median 98 vs 30 months; *P*=.02) [28). However, Falch et al [49] reported that tumor stage, complete tumor resection rate, and overall complication rate were not influenced by age, thus challenging the findings of the effect of age on survival. Significant effects of comorbidity and treatment choice were observed on the overall, disease-specific, progression-free survival, and disease-free survival rates. Functional status of patients was a predictor for survival in a study conducted by Sanabria et al [26], which reiterates its importance in treatment choice. No conclusive evidence regarding QoL and comorbidities could be obtained as one study showed significant association [50], and no significant association was identified in another study [22].

The studies from this systematic review indicated that physical comorbidities are extensively prevalent among older cancer patients and impact various treatment stages. However, the exact mechanism by which physical comorbidities impact treatment was not demonstrated by any article other than identifying a relationship between age and physical comorbidity. Therefore, the influence of physical comorbidity on treatment outcomes is still unknown, and this signifies the need for research to conclude how comorbidity impacts treatment and treatment outcomes in older cancer patients.

### Conclusions

With a growing population, the number of cancer cases is also on the rise. An increasing older population, as a proportion of the overall population, will also be reflected in a growing older cancer patient population, which contributes a significant proportion of the cancer patient population in general. Future large-scale, multicentered longitudinal randomized trials focused on the older adult population are therefore warranted to measure the effects of comorbidities on physical and psychological variables of interest in addition to QoL. Studies that test self-management interventions, such as exercise, are also needed to assess their impact on the management of comorbidities, and subsequent improvement of symptoms and functional status, thereby improving QoL for older patients with cancer. Additionally, integration of data related to symptoms into routine electronic records and care remains a high priority. These studies should include and stratify older patients by functional status, comorbid conditions, older adult syndromes, and prognosis to better represent the real-world population and improve research validity. Treatment of comorbidity, the severity of comorbidity, and the interaction of comorbidity with cancer treatment have not been discussed in the papers selected for this review. Overall, increased age and increased comorbidities correlate with significantly lesser likelihood of treatment initiation. Some variability in the included comorbidities and comorbidity scoring and the potential for other confounding variables (eg, marital status, as per Hu et al [45]) to complicate reported outcomes impact the statistical and clinical significance of this group of studies.

This systematic review provides evidence to prove the varied impact of physical comorbidities on cancer treatment and outcomes among older adult populations. It is suggested that comorbid older adult patients with better functional status might tolerate this treatment and show better survival and QoL outcomes when provided with standard and more aggressive treatment. Therefore, comprehensive older adult assessments are strongly recommended; they can help analyze the health status of older individuals, which then influences treatment decisions. Unfortunately, the quality of the majority of studies in this review was low, which makes incorporating their recommendations into routine practice less certain. Hence, this study recommends high-quality evidence generation in older adult cancer patients with physical comorbidities to translate research findings to clinical practice.

## Appendix

Multimedia Appendix 1 Search Strategy.

Multimedia Appendix 2 Supplementary Table 1: Summary of included studies.

Multimedia Appendix 3 Supplementary Table 2: Characteristics and quality of studies selected for review on impact of comorbidity in older adult cancer treatment.

Multimedia Appendix 4 Supplementary Tables 3 and 4.

## Abbreviations

AOR: adjusted odds ratio
ASCO: American Society of Clinical Oncology
CCI: Charlson Comorbidity Index
CHF: congestive heart failure
COPD: chronic obstructive pulmonary disease
EORTC: European Organization for Research and Treatment of Cancer Quality of Life Questionnaire
GA: geriatric assessment
HR: hazard ratio
OR: odds ratio
OS: overall survival
PRISMA: preferred reporting items for systematic reviews and meta-analysis
QoL: quality of life
