# Supplementary material for: Physical Comorbidities and Their Relationship with Cancer Treatment and Its Outcomes in Older Adult Populations: Systematic Review
Source: JMIR Cancer. 2021 Oct 13;7(4):e26425. doi: 10.2196/26425 (PMC8552093; doi:10.2196/26425)
Supplement: Multimedia Appendix 1 [file cancer_v7i4e26425_app1.docx]

***Search strategy for inclusion of studies***

| Embase | | | PubMed | | | Google Scholar | |
| --- | --- | --- | --- | --- | --- | --- | --- |
| Indexed Search terms (Emtree) | **Free text words** | **Search strategy** | **Indexed Search terms** | **Free text words** | **Search strategy** | **Free text words** | **Search Strategy** |
| [Geriatric cancer]  Exp | Elderly cancer OR  Old age cancer OR Geriatric oncology | [Geriatric cancer]  exp OR Elderly cancer OR  Old age cancer OR Geriatric oncology | Geriatric cancer [MeSH] | Elderly cancer OR  Old age cancer OR Geriatric oncology | Geriatric cancer [MeSH] OR  Elderly cancer OR  Old age cancer OR Geriatric oncology | Geriatric cancer OR  Elderly cancer OR  Old age cancer OR Geriatric oncology | Geriatric cancer OR  Elderly cancer OR  Old age cancer OR Geriatric oncology |
|  |  | AND |  |  | AND |  | AND |
| [Cancer treatment]  Exp | Surgery OR  Adjuvant OR  Palliative OR  Neo adjuvant OR  Concurrent OR  Chemotherapy OR  Radiation therapy | [Cancer treatment]  exp OR Surgery OR  Adjuvant OR  Palliative OR  Neo adjuvant OR  Concurrent OR Chemotherapy OR  Radiation therapy | Cancer treatment [MeSH] | Surgery OR Adjuvant OR Palliative OR  Neo adjuvant OR  Concurrent OR Chemotherapy OR  Radiation therapy | Cancer treatment [MeSH] OR  Surgery OR Adjuvant OR Palliative OR  Neo adjuvant OR  Concurrent OR Chemotherapy OR  Radiation therapy | Cancer treatment OR  Surgery OR Adjuvant OR Palliative OR  Neo adjuvant OR  Concurrent OR Chemotherapy OR  Radiation therapy | Cancer treatment OR  Surgery OR Adjuvant OR Palliative OR  Neo adjuvant OR  Concurrent OR Chemotherapy OR  Radiation therapy |
|  |  | AND |  |  | AND |  | AND |
| [Physical comorbidity]  Exp | Comorbidity OR Epidemiological factors OR Diabetes mellitus OR Hypertension OR chronic kidney disease OR Gastrointestinal disease OR Obesity OR Cardiovascular disease OR Pulmonary disease OR Neurological disease OR Respiratory disease OR Thyroid OR Liver disease | [Physical comorbidity]  exp OR  Comorbidity OR Epidemiological factors OR Diabetes mellitus OR Hypertension OR chronic kidney disease OR Gastrointestinal disease OR Obesity OR Cardiovascular disease OR Pulmonary disease OR Neurological disease OR Respiratory disease OR Thyroid OR Liver disease | Physical comorbidity [MeSH] | Comorbidity OR Epidemiological factors OR Diabetes mellitus OR Hypertension OR chronic kidney disease OR Gastrointestinal disease OR Obesity OR Cardiovascular disease OR Pulmonary disease OR Neurological disease OR Respiratory disease OR Thyroid OR Liver disease | Physical comorbidity [MeSH] OR Comorbidity OR Epidemiological factors OR Diabetes mellitus OR Hypertension OR chronic kidney disease OR Gastrointestinal disease OR Obesity OR Cardiovascular disease OR Pulmonary disease OR Neurological disease OR Respiratory disease OR Thyroid OR Liver disease | Physical comorbidity OR Comorbidity OR Epidemiological factors OR Diabetes mellitus OR Hypertension OR chronic kidney disease OR Gastrointestinal disease OR Obesity OR Cardiovascular disease OR Pulmonary disease OR Neurological disease OR Respiratory disease OR Thyroid OR Liver disease | Physical comorbidity OR Comorbidity OR Epidemiological factors OR Diabetes mellitus OR Hypertension OR chronic kidney disease OR Gastrointestinal disease OR Obesity OR Cardiovascular disease OR Pulmonary disease OR Neurological disease OR Respiratory disease OR Thyroid OR Liver disease |
|  |  | AND |  |  | AND |  | AND |
| [Treatment outcomes] | Clinical outcome OR disease free interval OR outcome assessment OR partial drug response OR patient reported outcome OR treatment failure OR therapeutic outcome OR therapy outcome OR treatment effect OR  Treatment withdrawal OR  Treatment delay OR drug carcinogenicity OR drug cytotoxicity OR toxicity | [Treatment outcomes] OR Clinical outcome OR disease free interval OR outcome assessment OR partial drug response OR patient reported outcome OR treatment failure OR therapeutic outcome OR therapy outcome OR treatment effect OR  Treatment withdrawal OR  Treatment delay OR drug carcinogenicity OR drug cytotoxicity OR toxicity | [Treatment outcomes] | Therapy OR treatment OR therapeutics [MeSH] OR Clinical outcome OR disease free interval OR outcome assessment OR partial drug response OR patient reported outcome OR treatment failure OR therapeutic outcome OR therapy outcome OR treatment effect OR  Treatment withdrawal OR  Treatment delay OR drug carcinogenicity OR drug cytotoxicity OR toxicity | [Treatment outcomes] OR Therapy OR treatment OR therapeutics [MeSH] OR Clinical outcome OR disease free interval OR outcome assessment OR partial drug response OR patient reported outcome OR treatment failure OR therapeutic outcome OR therapy outcome OR treatment effect OR  Treatment withdrawal OR  Treatment delay OR drug carcinogenicity OR drug cytotoxicity OR toxicity | Treatment OR Initiation OR Completion OR delay OR toxicity | Therapy OR treatment OR therapeutics OR Initiation OR Completion OR adverse effects OR Complication OR Clinical outcome OR disease free interval OR outcome assessment OR partial drug response OR patient reported outcome OR treatment failure OR therapeutic outcome OR therapy outcome OR treatment effect OR  Treatment withdrawal OR  Treatment delay OR drug carcinogenicity OR drug cytotoxicity OR toxicity |
|  |  | AND |  |  | AND |  | AND |
| [Survival OR Quality of life] exp | Mortality OR  Death OR  Physical wellbeing OR  Wellbeing OR Psychological function OR Physical function OR Functional ability OR Fatigue OR Pain OR Sleep OR Frailty | [Survival OR Quality of life] exp OR  Mortality OR  Death OR  Physical wellbeing OR  Wellbeing OR Psychological function OR Physical function OR Functional ability OR Fatigue OR Pain OR Sleep OR Frailty | Survival OR Quality of life [MeSH] | Mortality OR  Death OR  Physical wellbeing OR  Wellbeing OR Psychological function OR Physical function OR Functional ability OR Fatigue OR Pain OR Sleep OR Frailty | Survival OR Quality of life [MeSH] OR  Mortality OR  Death OR  Physical wellbeing OR  Wellbeing OR Psychological function OR Physical function OR Functional ability OR Fatigue OR Pain OR Sleep OR Frailty | Survival OR Quality of life OR Mortality OR  Death OR  Physical wellbeing OR  Wellbeing OR Psychological function OR Physical function OR Functional ability OR Fatigue OR Pain OR Sleep OR Frailty | Survival OR Quality of life OR Mortality OR  Death OR  Physical wellbeing OR  Wellbeing OR Psychological function OR Physical function OR Functional ability OR Fatigue OR Pain OR Sleep OR Frailty |

*Note*: exp - Explode
