# Supplementary material for: Physical Comorbidities and Their Relationship with Cancer Treatment and Its Outcomes in Older Adult Populations: Systematic Review
Source: JMIR Cancer. 2021 Oct 13;7(4):e26425. doi: 10.2196/26425 (PMC8552093; doi:10.2196/26425)
Supplement: Multimedia Appendix 2 [file cancer_v7i4e26425_app2.docx]

## Supplementary Table 1: Summary of included studies

| Description | Categories | Number (N=29) |
| --- | --- | --- |
| Country of Research | | |
|  | USA | 11 |
|  | Netherlands | 5 |
|  | Germany | 3 |
|  | Italy | 1 |
|  | Norway | 1 |
|  | Denmark | 1 |
|  | Sweden | 1 |
|  | Portugal | 1 |
|  | Brazil | 1 |
|  | Singapore | 1 |
|  | Thailand | 1 |
|  | India | 1 |
|  | New Zealand | 1 |
| Cancer site | | |
|  | Colon | 7 |
|  | Head and neck | 5 |
|  | Breast | 4 |
|  | Lung | 3 |
|  | Colorectal | 3 |
|  | Ovary | 2 |
|  | Hematological | 2 |
|  | Mixed | 2 |
|  | Rectum | 1 |
| Sample size Description | | |
|  | Less than 100 | 3 |
|  | 101-200 | 3 |
|  | 201-300 | 2 |
|  | 301-500 | 3 |
|  | 501- 700 | 5 |
|  | 701- 1000 | 3 |
|  | Above 1000 | 10 |
| Study type | | |
|  | Record review | 6 |
|  | Retrospective | 5 |
|  | Cohort | 2 |
|  | Prospective | 1 |
|  | Cross sectional | 1 |
|  | Prospective cohort | 1 |
|  | Retrospective cohort | 1 |
|  | Not mentioned | 12 |
| Gender | | |
|  | Male | 1 |
|  | Female | 5 |
|  | Both | 23 |
| Treatment | | |
|  | Chemotherapy | 12 |
|  | Surgery+ Chemo | 6 |
|  | Surgery | 5 |
|  | Surgery+ RT+ CT | 3 |
|  | RT+CT | 1 |
|  | Surgery+ RT | 1 |
|  | NM | 1 |
|  |  |  |
| Year of publication | | |
|  | 1991-1995 | 0 |
|  | 1996-2000 | 1 |
|  | 2001-2005 | 5 |
|  | 2006-2010 | 9 |
|  | 2011-2015 | 12 |
|  | 2016-2018 | 2 |
| Number of Co-morbidities reported | | |
|  | Less than 2 | 9 |
|  | 3-5 | 7 |
|  | More than 5 | 9 |
|  | Any comorbidity | 4 |
| Co-morbidities reported | | |
|  | Diabetes | 12 |
|  | Hypertension | 10 |
|  | Cerebrovascular disease | 9 |
|  | Cardiovascular/pulmonary disease | 8 |
|  | Heart Failure | 8 |
|  | Myocardial infarction | 7 |
|  | Dementia | 6 |
|  | Peripheral Vascular disease | 6 |
|  | Paralysis | 5 |
|  | Respiratory disease | 5 |
|  | Liver | 5 |
|  | Renal disease | 4 |
|  | HIV/AIDS | 4 |
|  | Ulcer | 4 |
|  | Gastro intestinal disease | 3 |
|  | Obesity | 3 |
|  | Hematopoietic | 3 |
|  | Psychiatric problem | 3 |
|  | Apoplexy | 2 |
|  | Thromboembolism | 2 |
|  | Arthritis | 1 |
|  | Lung | 1 |
|  | Neuromuscular | 1 |
|  | Poor hearing sight | 1 |
|  | Poor eye sight | 1 |
|  | Type of co morbidity not mentioned | 6 |
| Outcome | | |
|  | Survival +Treatment Initiation | 12 |
|  | Treatment | 5 |
|  | QOL | 3 |
|  | Survival | 3 |
|  | Survival+QOL | 2 |
|  | Overall Survival | 2 |
|  | QOL+Treatment | 2 |
