# Supplementary material for: Physical Comorbidities and Their Relationship with Cancer Treatment and Its Outcomes in Older Adult Populations: Systematic Review
Source: JMIR Cancer. 2021 Oct 13;7(4):e26425. doi: 10.2196/26425 (PMC8552093; doi:10.2196/26425)
Supplement: Multimedia Appendix 3 [file cancer_v7i4e26425_app3.docx]

## Supplementary Table 2: Characteristics and quality of studies selected for review on impact of comorbidity in older adult cancer treatment

| S. No. | Author | | Year | Country | Setting | Design | No. of patients | Sex | Age in years  Mean (SD)  [Median(Range)] | Cancer site | Stage | Treatment - % | Tools Used | Quality of Assessment |
| --- | --- | --- | --- | --- | --- | --- | --- | --- | --- | --- | --- | --- | --- | --- |
|  | | BREAST | | | | | | | | | | | | |
|  | Berglund et al | | 2012 | Sweden | Hospital Records | Retrospective | 42,646 | F | All age group | Breast | I, IIA, IIB, III, IV | Sx, CT, RT- NM | CCI | Low |
|  | Klepin et al | | 2014 | USA | Hospital Records | Retrospective | 329 | F | 71(65-89) | Breast | I,II,III | Adj. ±CT- NM | OARS | Moderate |
|  | O'Connor et al | | 2012 | USA | Registry based | Retrospective | 204 | F | 70(65–86) | Breast | I,II,III | NACT-23%  NART- 86%  Sx- 98% | CCI | Low |
|  | Houterman et al | | 2004 | Netherlands | Registry based | Retrospective | 527 | F | 40-69 &  ≥70 | Breast | NM | Sx -25%  CT-1%  RT- 38%  HT- 44% | Medical Records | Low |
|  | | COLORECTAL | | | | | | | | | | | | |
|  | Gross et al | | 2007 | USA | Registry based | Retrospective | 5330 | M&F | Median -76 | Colon | III | ACT- 60.3% | CCI | High |
|  | Koroukian | | 2009 | USA | Registry based | Retrospective | 957 | M&F | 77.6(± 7.0) | Colorectal | NM | Sx,- 89%  CT- 36% | ADL | Low |
|  | Hu et al | | 2011 | USA | Registry based | Retrospective cohort | 12,265 | M&F | 65+ | Colon | III | Sx±Adj. CT- NM | Self designed | Low |
|  | Sarfati et al | | 2009 | New Zealand | Registry based | Retrospective cohort | 589 | M&F | 55+ | Colon | I, II, III, IV | Adj. ±CT- NM | CCI | Low |
|  | Lemmens et al | | 2005 | Netherlands | Hospital Records | Retrospective | 577 | M&F | 76.5 (65-79) | Colon | IIIA, IIIB,IIIC | CT- 83% | CCI | Low |
|  | Sanoff et al | | 2012 | USA | Registry based | Retrospective | 5489 | M&F | ≥75 | Colon | III | CT (SEER-Medicare (42%), and  NYSCR-Medicare (28%), than at NCCN centers (61%). | ACE-27 | Low |
|  | Falch et al | | 2009 | Germany | Hospital Records | Retrospective | 394 | M&F | 83.8 ± 3.6 | Colorectal | I, II, III, IV | Sx- 94% | NM | Low |
|  | Miguel et al | | 2015 | Portugal | Hospital Records | Retrospective | 59 | M&F | 79.9 (± 3.8) | Rectum | II,III | NACT- 79.7%  RT- 20.3%  Sx- 89.8% | CCI | Moderate |
|  | Tan et al | | 2012 | Singapore | Hospital Records | Retrospective | 204 | M&F | 84  (80–97) | Colorectal | I, II, III, IV | Sx- 91.7% | CCI  ASA score | Low |
|  | Hoeben et al | | 2013 | Netherlands | Registry based | Retrospective | 557 | M | 82  (75–95) | Colon | III | CT-27% | ACE-27 | Moderate |
|  | Koroukian et al | | 2010 | USA | Registry based | Retrospective | 1,009 | M&F | NM  (65–85) | Colon, Rectum | I,II | Sx, ±CT- 31.4%  Sx- 53.2%  Palliative care- 15.4% | Vital records | Low |
|  | Sundararajan et al | | 2001 | USA | Registry based | Retrospective | 4998 | M&F | 76 (65-103) | Colon | III | CT -50% | Deyo -CCI | Low |
|  | | HEAD & NECK | | | | | | | | | | | | |
|  | Derks et al | | 2005 | Netherlands | Hospital records | Cross-sectional | 266 | M&F | 45-60  70-79  ≥80 | Head and Neck | II,III, IV | CT, RT, Sx – NM | EORTCQLQ-C30, H&N35,  CES-D, KFI, RSS 12-I, QQ | Low |
|  | Sanabria et al | | 2007 | Brazil | Hospital Records | Retrospective | 310 | M&F | 76.0  (±5.3 Median 75(70-93) | Head and Neck | I, II, III, IV | ±CT, ±RT, Sx - NM | ACE-27 | Moderate |
|  | Genther & Gourin | | 2015 | USA | Registry based | Retrospective | 61,740 | M&F | 73  (66-104) | Head and Neck | NM | Sx- NM | CCI | Low |
|  | Peters et al | | 2015 | Netherlands | Registry based | Retrospective | 202 | M&F | 70+ | Head & Neck | I, II, III, IV | Sx- (n=202) | ACE-27 | Low |
|  | | LEUKEMIA | | | | | | | | | | | | |
|  | Goede et al | | 2014 | Germany and Austria | Hospital Records | Prospective | 581 | M&F | 74 (48-87) | Leukemia (CLL) | - | CT- 73% | CCI | Low |
|  | | LUNG | | | | | | | | | | | | |
|  | de Rijke et al | | 2004 | Netherlands | Registry  based | Retrospective | 803 | M&F | All ages± | Lung | I,II, IIIA, IIIB,IV | Sx, RT, CT- NM | CCI, KFI | Low |
|  | Gronberg et al | | 2010 | Norway | Hospital records | Retrospective | 402 | M&F | 65(25-90) | Lung | III,IV | CT- 65% | CIRS-G, EORTC QLQC30, LC13 | Moderate |
|  | Pathy et al | | 2016 | India | Hospital Records | Retrospective | 96 | M&F | 72(65-82) | Lung | IIIa,,IIIb | NACT- 50%  RT-67%  Palliative RT- 71%  con.CTRT- 33% | Self Reported | Low |
|  | | MIXED TYPES | | | | | | | | | | | | |
|  | Phaibulvatanapong et al | | 2018 | Thailand | Hospital Records | Prospective | 151 | M&F | 76.37 (70.0-88.1) | Mixed | NM | CT- 62.9% | CCI, FI, SGA, FACT –G, MMSE | Low |
|  | Piccirillo | | 2000 | USA | Hospital Records | Prospective cohort | 3378 | M&F | Mean 62.8(±12.6) Median 77(18-98) | Mixed | I, II, III, IV | Sx-88%  RT- 33%  Combined therapy- 53% | KFI | Low |
|  | Koroukian et al | | 2011 | USA | Registry based | Retrospective | 1236 | M&F | NM  (65–85) | Mixed | I,II | Sx ±CT±RT Standard treatment- NM | NIA/NCI code | Low |
|  | | OVARIAN | | | | | | | | | | | | |
|  | Jorgensen et al | | 2012 | Denmark | Registry based | Retrospective cohort study | 961 | F | 74  (61-82) | Ovary | I, II, III, IV | Sx, ±CT- NM | CCI  ASA score | Low |
|  | Ferrero et al | | 2017 | Italy | Hospital Records | Retrospective multicenter study | 78 | F | 75.6  (70.89) | Ovary | I, II, III, IV | CT, Sx- NM | mFI | Low |

NM- Not mentioned. M(MR)- Median (Median Range). x(SD) – Mean (Standard Deviation). NM – Not mentioned. CIRS-G Cumulative Illness Rating scale for Geriatrics, EORTC QLQC30 European Organisation for Research and Treatment of Cancer Quality of Life Questionnaire., EORTC LC13 - EORTC Lung Cancer Quality of Life Questionnaire. CCI – Charlson Comorbidity Index. FI- Frailty Index. SGA Subjective Global Assessment. FACT –G Functional Assessment of Cancer Therapy -General. MMSE- Mini-mental State Examination. ADL – Activities of Daily Life. KFI- Kaplan-Feinstein index. EORTC QOL (H&N35) – EORTC Head and Neck Cancer Quality of Life Questionnaire. CES-D Center for Epidemiological studies depression Inventory, RSS 12-I – Social Support List-interactions. QQ – Quality-Quantity questionnaire. NCI/NIA – National Cancer Institute/national Institute on Aging. ACE-27- Adult Comorbidity Evaluation Index. ASA- American Society of Anesthesiologists. mFI- Modified Frailty Index. Sx- Surgery. CT – Chemotherapy. RT- Radiotherapy. NACT – Neoadjuvant chemotherapy. Adj.- Adjuvant. Con. – CTRT – Chemoradiotherapy, OARS- Older Americans Resources and Services Questionnaire
