# Supplementary material for: Physical Comorbidities and Their Relationship with Cancer Treatment and Its Outcomes in Older Adult Populations: Systematic Review
Source: JMIR Cancer. 2021 Oct 13;7(4):e26425. doi: 10.2196/26425 (PMC8552093; doi:10.2196/26425)
Supplement: Multimedia Appendix 4 [file cancer_v7i4e26425_app4.docx]

## Supplementary Table 3: Impact of comorbidity on treatment outcomes of elderly cancer patients and other salient findings

| S.No. | Article code | Author (Year) | Comorbidity Details | Treatment-related impacts | | | | | | Other key findings |
| --- | --- | --- | --- | --- | --- | --- | --- | --- | --- | --- |
|  |  |  |  | **Initiation** | **Dose altered** | **Delay or discontinuation** | **Completion** | **Choice of Treatment** | **Toxicity/Adverse effect/ Post-operative complications** |  |
| 1 | D_1 | de Rijke et al (2004) | No. of comorbidities  0-57.3%  1-85.6%  2-89.3%  ≥ 3-96% |  |  |  |  | Stage I & II  +(Covariates – age +)  Stage IIIA  #(Covariates – age+)  Stage IIIB  # (Covariates – age#) |  | a.Comorbidities increased with age while FS decreased  b. After adjusting for age and stage, comorbidity and FS were correlated only with localized stages  c. Comorbidity significantly affected Tx choice for localized stage  d. Chronological age was considered while Tx decision making |
| 2 | D_10 | Gross et al (2007) | No. of comorbidities  0-46.5%  1-2 44.7%  ≥ 3 -8.8% | + |  |  | # | +  Covariates – age, marital status + | Toxicity related hospitalization # | a.COPD, heart failure and DM were the most commonly reported comorbidities  b. Heart failure was significantly associated while COPD and DM were moderately associated with receiving adjuvant CT  c. Adjuvant therapy yielded better results in patients without CHF, COPD and DM than those who suffered these conditions.  d.Comorbid patients were more hospitalized than those without comorbidity  e. CT may increase the toxicity risk in patients without CHF than in those with CHF while no significant association was found between COPD or DM with adjuvant CT |
| 3 | D_9 | Gronberg et al (2010) | Severity  0-No-0.7%  >1- 8%  >2- 15%- high severity index  3-4 49% - severe comorbidity  4- 9% - extremely severe comorbidity  (No severe comorbidity-51.2%  Severe comorbidity – 48.8%) |  | #* |  | +* | # | Toxicity + | Respiratory, vascular and heart diseases were the most commonly reported comorbidities |
| 4 | A_78 | Phaibulvatanapong et al (2018) | Comorbidity score  2-3 -NM  4-5 0.7%  6-7 31.1%  >8 68.2% |  |  | Discontinuation +  Covariates – disease progression and toxicity + |  |  | #  Covariates – FS, Tx choice, BMI +  Frailty, cognitive function and family support # | a. 2/3^rd^ of patients suffered from comorbidities; despite good FS, many participants were classified into frail and elderly  b. CCI can’t be used to assess comorbidity in cancer since CCI will mostly show higher score for metastatic patients |
| 5 | J_97 | Koroukian et al (2009) | NM |  |  |  |  | +  Covariates – Limitation of ADL, GS + |  | a. Comorbidity along with limitation in ADL resulted in not receiving Tx  b.FL and GS are important in geriatric cancer management |
| 6 | J_104 | Derks et al (2005) | No-25%  Mild-28%  Moderate-36%  Severe-10% |  |  |  |  | +  Covariates (for 45-70 yrs) – Marital state, tumour grade, age, patient preference +  Covariates (>70 yrs)-age, stage, site + |  | a.Even after adjusting comorbidity age was independently associated with Tx choice  b. Patient preference affected Tx choice – 18% of the age group ≥ 80 yrs refused Tx proposal |
| 7 | J_113 | Piccirillo et al (2000) | None 56.1%  Mild 23.5%  Moderate 15.1%  Severe  5.2% |  |  |  |  |  |  | Patients with high comorbidity treated with combined therapy can provide better outcomes |
| 8 | D_115 | Sanabria et al (2007) | Severity  0-24.9%  1-45.6%  2-15.5%  3-13.9% |  |  |  |  |  |  | a.Patients did not receive Tx due to death or preference  b. HT followed by respiratory disease and cognitive heart failure were the most commonly reported comorbidities |
| 9 | J_46 | Genther & Gourin (2015) | Cormorbidity score  0-1 81%  2-13%  ≥3-6% |  |  |  |  | + | Post-operative complications #  Acute medical complications + (covariates – age, emergency admission +) | a.CVD followed by CPD were the most commonly reported comorbidities  b.Patients with high comorbidity and > 75 yrs had increased risks of acute events  c. Comorbidity is an independent predictor for risk of in-hospital death  d. Extent of surgery but not comorbidity was related to length of hospitalization and hospital- related cost |
| 10 | A_13 | Hu et al (2011) | Cormorbidity score  0-52.2%  1-28.1%  ≥2-19.6% | +  Covariates-age at diagnosis, marital status, recent yr of diagnosis + |  |  | +  Covariates-age at diagnosis, marital status, recent yr of diagnosis +  SES and residence # |  |  | Age at diagnosis (Strongest predictor), comorbidity, marital status were predictors for Tx initiation and completion |
| 11 | D_2 | Sarfati et al (2009) | CCI score  0-48.2%  1-2 40.8%  3+ 11.0%  No. of comorbidities  0-30.7%  1-25%  2-19.9%  3+ 24.5% |  |  |  |  | +  Covariates – Age+ |  | a.70% had at least one comorbid condition  b. Stage III patients had high comorbidity |
| 12 | D_6 | Berglund et al (2012) | Severity  0-no -87.4%  1-mild 6.6%  2+-severe 6.0 % |  |  |  |  | + |  | a. Mastectomy not associated with comorbidity  b. Breast conserving surgery, radiation and CT offered less in the presence of high comorbidity (less extensive Tx provided) |
| 13 | J_23 | Lemmens et al (2005) | No. of comorbidities  0-31.9%  1-33.6%  2+ 27.6% |  |  |  |  | +  Covariates – Age, Gender, SES, tumour grade + |  | COPD was significantly associated with not receiving Tx |
| 14 | F_34 | Goede et al (2014) | No. of comorbidities  0-47%  1-28%  ≥2-25%  53% had at least one comorbidity |  | + |  | +  Covariates – age, treatmetn # |  | #  Covariates – age,Tx # | a.Metabolic/endocrine followed by vascular and cardiac disease were the highest reported comorbidities  b. Lower Tx response in paients with high comorbidity score but no difference after adjusting for age and Tx  c. No relation was found between toxicity and individual comorbidity  d. Increased prognosis due to comorbidity along with dose reduction and Tx discontinuation  e.Comorbidity is an independent predictor for adverse outcomes |
| 15 | F_37 | Jorgensen et al (2012) | 0-71.7%  1+ 23.7% (according to CCI)  1-36.2%  2-45.2%  3+ 17.9%  (according to ASA score) |  | + | Discontinuation ∑  Covariates – performance status ∑ |  | ∑ |  | a.Age was independent predictor for not receiving Tx  b. Comorbidity but not age, was a prognostic factor for patients receiving standard combination CT  c. Tx not provided due to poor FS or patient preference  d. Disease prognosis was more in >70 yrs due to comorbidity, advanced stage at diagnosis, performance status |
| 16 | A_67 | Peters et al (2015) | None  Mild  Moderate Severe  (n % NM) |  |  |  |  |  | Medical Complications +  ∑ | Biological age instead of chronological age should be considered for Tx choice |
| 17 | A_69 | Sanoff et al (2012) | No. of comorbidities  SEER, NYSCR, NCCN  0-52.7%, 67.2%, 47.9%  1-27.2%, 18.8%, 51.4%  >2-20.1%, -, - |  |  |  |  | +  Covariate – Age + |  |  |
| 18 | A_73 | Ferrero et al (2017) | NM  Highest reported comorbidity- Hypertension 49.4% |  |  | Delay #  Discontinuation #  Covariates for both – Age # | +* | # | Surgical complications +  Toxicity # | a.HT followed by CVD, DM and CRF were the most commonly reported comorbidities in both 70 to 75 yrs and > 75 yrs group  b. High-frailty group had poor FS  c. Age and frailty independently associated with Tx response  d. Chronological age alone should not be considered for Tx choice |
| 19 | A_75 | Falch et al (2009) | No. of comorbidities  0-24.6%  1-36.8%  2-22.3%  3-13.7%  ≥4- 2.5% |  |  |  |  |  |  | a.More comorbid conditions above 80 yrs than in 69-70 yrs group  b. Patients did not receive Tx due to CVD  c. CVD followed by HT and DM were the most commonly reported comorbidities |
| 20 | D_84 | Miguel et al (2015) | Severity  0-1 Fit- 72.9%  ≥2 – Vulnerable- 27.1% |  | #* |  |  | #*  Covariates – age #* | +* | a. Refusal to undergo Tx by patients  b. CVD followed by CPD-most commonly reported |
| 21 | D_85 | Tan et al (2012) | CCI  ≤3 62.7%  >3- 37.3%  Median – 3(2-10)  ASA score  2-30.4%  3-63.2%  4-6.4% |  |  |  |  | +  Covariates – age, patient preference |  | a. Age and comorbidity significantly associated with severe grades of perioperative outcomes  b. Reluctance of patients to undergo post-operative investigations was observed  c. HT followed by DM and ischemic heart disease was the most commonly reported |
| 22 | D_86 | Hoeben et al (2013) | Severity  No-25.9%  Mild-29.1%  Moderate-24.8%  Severe-17.8%  3/4^th^ of patients had comorbidities at the time of diagnosis |  |  | Delay #  Covariates – Age # |  |  | Toxicity #  Covariates – Age # | a.3/4^th^ of study population had comorbidity at the time of diagnosis  b. Age, comorbidity, performance status and denial by patient were causes for not receiving Tx  c.CVD followed by DM and pulmonary disease were the most common comorbidities |
| 23 | D_87 | Klepin et al (2014) | No. of comorbidities  Median- 2 (0-10)  Comorbidity burden score  Median 3(0-25) |  | + | Discontinuation + |  |  | # | a.Arthiritis followed by HT were the most common comorbities  b. Dose alteration was associated with comorbidity and not toxicity  c. Comorbidity was not related to time of relapse |
| 24 | J_98 | Koroukian et al (2010) | No. of comorbidities  0-28.8%  1-29.4%  >2-41.8% |  |  |  |  | +  Covariates– FL,GS, age, race, stage + |  | Effect of comorbidity was attenuated by age |
| 25 | J_99 | Koroukian et al (2011) | Multimorbidity – comorbidities, FL, GS  0-21.2%  1-36.3%  2-30%  3-12.5% |  |  |  |  | +  Covariates – age+ |  |  |
| 26 | J_100 | Pathy et al (2016) | No. of cormorbidities  0-1 41%  ≥2 - 59% |  |  |  |  |  |  | Old pulmonary TB, COPD, HT and DM were the most commonly reported comorbidities |
| 27 | J_101 | O' Connor et al (2012) | Comorbidity score  0-51%  ≥1- 49% |  |  | Delay+  Covariates- Tx choice +  Discontinuation+  Covariates-Age |  |  |  | a.Age was a predictor for Tx discontinuation, RDI and less aggressive Tx  b.HT correlated with Tx delay and hospitalization |
| 28 | J_117 | Houterman et al (2004) | No comorbidity-24%  Low Impact- 35.3%  Moderate Impact- 42%  High Impact-46% |  |  |  |  | #  Covariate – age + | Complication #  Covariates- Age # | a.Older patients with severe comorbidity showed worse prognosis  b. Most common conditions reported were HT, CVD and DM |
| 29 | J_119 | Sundararajan et al (2001) | No. of comorbidities  0-60.7%  1-25.2%  >1-11.7% |  |  |  |  | +  Covariate – age, no. of nodes, year of diagnosis +  Gender # |  |  |

*Statistically not significant. + significant association. # no significant association. ∑ Predictor. FL – Functional Limitations. GS- Geriatric syndromes. RDI-Relative Dose Intensity. SES – Socioeconomic status. Tx-Treatment. FS- functional status. CHF- Congestive Heart Failure. COPD – Chronic Obstructive Pulmonary Disorder. HT- Hypertension. DM- Diabetes Mellitus. CVD – Cardiovascular Disease. TB- Tuberculosis. CCI- Charlson Comorbidity Index. CPD – Chronic Pulmonary Disease. CRF – Chronic Renal Failure. CT - Chemotherapy

## Supplementary Table 4: Influence of comorbidity on quality of life and survival of older adult cancer patients and other salient findings

| S.No. | Article code | Author (Year) | Comorbidity Details | Quality of life | Survival | Covariates of Survival | Other key findings |
| --- | --- | --- | --- | --- | --- | --- | --- |
| 1 | D_1 | de Rijke et al (2004) | No. of comorbidities  0-57.3%  1-85.6%  2-89.3%  ≥ 3-96% |  | NIL |  |  |
| 2 | D_10 | Gross et al (2007) | No. of comorbidities  0-46.5%  1-2 44.7%  ≥ 3 -8.8% |  | + |  | a.Mortality reduced significantly after receiving adjuvant CT (after adjusting SES and clinical characteristics)  b.Survival was similar for patients with and without CHF  c.Among patients without CHF, COPD or DM,5yr survival was greater in patients who received Tx than those who did not |
| 3 | D_9 | Gronberg et al (2010) | Severity  0-No-0.7%  >1- 8%  >2- 15%- high severity index  3-4 49% - severe comorbidity  4- 9% - extremely severe comorbidity  (No severe comorbidity-51.2%  Severe comorbidity – 48.8%) | + | OS #* | OS- Tx choice #* | a.Physical and role functioning in HRQoL scales was poor in patients with high comorbidity  b. Sensitivity test confirmed global QoL but not fatigue, nausea and vomiting in patients with severe comorbidity |
| 4 | A_78 | Phaibulvatanapong et al (2018) | Comorbidity score  2-3 -NM  4-5 0.7%  6-7 31.1%  >8 68.2% | # |  |  | a.QoL at baseline for patients of FS score 2 was significantly poor than those with better FS  b. Comorbidity component of FACT-G showed insignificant reduction at the end of the study |
| 5 | J_97 | Koroukian et al (2009) | NM |  | OS #  CSS + | OS- GS, limited ADL +  CSS- GS+ | Comorbidities may not be associated with survival in the absence of FL and GS |
| 6 | J_104 | Derks et al (2005) | No-25%  Mild-28%  Moderate-36%  Severe-10% |  |  |  | a.Lower QoL in patients who did not receive standard Tx was observed  b.Pain component in QLQ C-30 was found be significantly associated with receiving non-standard Tx |
| 7 | J_113 | Piccirillo et al (2000) | None 56.1%  Mild 23.5%  Moderate 15.1%  Severe  5.2% |  | OS +  2 yr survival + | 2 yr survival – stage +  Initial Tx # | a.Patients with moderate comorbidity had two fold increased risk of mortality than that of no comorbidity  b. Strong risk was found for mortality > 70 yrs with moderate or severe comorbidity  c. Comorbidity was a prognostic factor (after adjusting for tumour grade and initial Tx) which shows that mortality is not only due to Tx effects |
| 8 | D_115 | Sanabria et al (2007) | Severity  0-24.9%  1-45.6%  2-15.5%  3-13.9% |  | OS +∑  CSS #∑ | OS – age, gender,FS, stage, site +  CSS – age, FS, stage | a.Comorbidity ≥ 2 increased the risk of death by 1.72 times  b. Comorbidity not associated with recurrence  c. FS was the strongest predictor for overall and specific survival  d. Age and FS associated with recurrence |
| 9 | J_46 | Genther & Gourin (2015) | Cormorbidity score  0-1 81%  2-13%  ≥3-6% |  | NIL |  |  |
| 10 | A_13 | Hu et al (2011) | Cormorbidity score  0-52.2%  1-28.1%  ≥2-19.6% |  | NIL |  |  |
| 11 | D_2 | Sarfati et al (2009) | CCI score  0-48.2%  1-2 40.8%  3+ 11.0%  No. of comorbidities  0-30.7%  1-25%  2-19.9%  3+ 24.5% |  | OS +  CSS + | OS, CSS- not being offered Tx, stage + (after adjusted for age, sex, comorbidity) | a.Congestive heart failure and non-cerebrovascular neurological conditions were associated to CSS  b. Individual comorbid conditions showed strong association with all-cause mortality then cancer specific mortality  c. Reduction in difference was found in survival between low and high comorbidity groups after adjusting for Tx.  d. All-cause and cancer specific mortality in patients with high comorbidity reduced by 60% when ‘offered CT’ |
| 12 | D_6 | Berglund et al (2012) | Severity  0-no -87.4%  1-mild 6.6%  2+-severe 6.0 % |  | CSS +  CBC +  CCM +  ACM + | CCM, CBC - stage | a.ACM duet to high comorbidity was 5 times more in 1^st^ yr and 2 times more in 10 yrs post-diagnosis  b. Comorbidity affected with CSS in early stages of breast cancer |
| 13 | J_23 | Lemmens et al (2005) | No. of comorbidities  0-31.9%  1-33.6%  2+ 27.6% |  | OS + | OS – age, disease stage +  Gender, SES and tumour grade # | After adjusting for covariates, receiving adjuvant CT reduced the mortality rate by half than those who did not receive it. |
| 14 | F_34 | Goede et al (2014) | No. of comorbidities  0-47%  1-28%  ≥2-25%  53% had at least one comorbidity |  | OS +  1yr and 5 yr mortality +  PFS |  | a. Higher PFS was related to Tx choice  b. Comorbidity was an independent prognostic factor for OS and independent determinant for PFS |
| 15 | F_37 | Jorgensen et al (2012) | 0-71.7%  1+ 23.7% (according to CCI)  1-36.2%  2-45.2%  3+ 17.9%  (according to ASA score) |  | OS +  PFS + | OS – age, surgical regimen + | a.Death due to prognosis and toxicity recorded  b. Effect of comorbidity score of 1 was a strong prognostic factor only upto 100 days (ASA score ≥1) in PFS and weak prognostic factor for OS until 100 days (ASA score ≥3) |
| 16 | A_67 | Peters et al (2015) | None  Mild  Moderate Severe  (n % NM) |  | OS, CSS # | OS, CSS – stage +  age # |  |
| 17 | A_69 | Sanoff et al (2012) | No. of comorbidities  SEER, NYSCR, NCCN  0-52.7%, 67.2%, 47.9%  1-27.2%, 18.8%, 51.4%  >2-20.1%, -, - |  |  | OS – Tx choice + | Patients > 75 yrs at stage III treated with adjuvant CT showed lower mortality rate than those who were not treated |
| 18 | A_73 | Ferrero et al (2017) | NM  Highest reported comorbidity- Hypertension 49.4% |  | OS + | OS –Tx choice, age + | OS decreased with age |
| 19 | A_75 | Falch et al (2009) | No. of comorbidities  0-24.6%  1-36.8%  2-22.3%  3-13.7%  ≥4- 2.5% |  | 30-day mortality +  5 yr survival + | 5 yr survival – tumour site  DFS - tumour site | a.Comorbidities significantly higher in above 80 age group than in 60-79 group.  b. Higher 30-day mortality, 5 yr survival in ≥80 age group  c. The difference in median DFS between two groups not statistically significant |
| 20 | D_84 | Miguel et al (2015) | Severity  0-1 Fit- 72.9%  ≥2 – Vulnerable- 27.1% |  | a. 1 yr survival #*  b. 3 yr survival and mean survival +  c. DFS #* |  | 1yr, 3yr and mean survival not affected by age, gender* |
| 21 | D_85 | Tan et al (2012) | CCI  ≤3 62.7%  >3- 37.3%  Median – 3(2-10)  ASA score  2-30.4%  3-63.2%  4-6.4% |  | # | 1. OS- disease stage, severe complication 2. DFS- disease stage, severe complication | a. Disease recurrence associated with peri-operative morbidity  b. Age, site of disease not related to survival |
| 22 | D_86 | Hoeben et al (2013) | Severity  No-25.9%  Mild-29.1%  Moderate-24.8%  Severe-17.8%  3/4^th^ of patients had comorbidities at the time of diagnosis |  | 5yr survival + | 5yr survival – Tx choice, age + | Patients who received CT showed better survival irrespective of age or toxicity |
| 23 | D_87 | Klepin et al (2014) | No. of comorbidities  Median- 2 (0-10)  Comorbidity burden score  Median 3(0-25) |  | OS + |  | Self-reported Chronic bronchitis, circulation trouble and stroke were related to high mortality |
| 24 | J_98 | Koroukian et al (2010) | No. of comorbidities  0-28.8%  1-29.4%  >2-41.8% |  | OS #  CSS + | a.OS- FL, GS+  b.CSS-GS+, FL# | In alternative models of FL only and GS only, comorbidities were not associated with CSS |
| 25 | J_99 | Koroukian et al (2011) | Multimorbidity – comorbidities, FL, GS  0-21.2%  1-36.3%  2-30%  3-12.5% |  | OS +  CSS # |  | CSS not related to receiving standard trreatment |
| 26 | J_100 | Pathy et al (2016) | No. of cormorbidities  0-1 41%  ≥2 - 59% |  | OS, PFS + | OS- SES, serum albumin, FS +  PFS - serum albumin, FS + |  |
| 27 | J_101 | O' Connor et al (2012) | Comorbidity score  0-51%  ≥1- 49% |  |  | OS-RDI and Tx discontinuation #  CSS- RDI and Tx discontinuation # | a.3 yr survival decreased in patients who discontinued Tx compared with those who completed Tx  b.After adjusting for age, comorbidity, biomarkers, stage and Tx, no relation was found between CSS with Tx discontinuation and RDI |
| 28 | J_117 | Houterman et al (2004) | No comorbidity-24%  Low Impact- 35.3%  Moderate Impact- 42%  High Impact-46% |  | 5 yr survival + | 5 yr survival - age + | After adjusting for age and Tx, risk of mortality was 2.4 times more in low- moderate comorbid patients and 2.9 times more in severe comorbid patients over 70 yrs |
| 29 | J_119 | Sundararajan et al (2001) | No. of comorbidities  0-60.7%  1-25.2%  >1-11.7% |  | NIL |  |  |

*Statistically not significant. + significant association. # no significant association. ∑ Predictor. FL – Functional Limitations. GS- Geriatric syndromes. RDI-Relative Dose Intensity. SES – Socioeconomic status. FS- functional status; CBC – Conditional Breast Cancer; CCM – Competing Cause Mortality; ACM –All-cause Mortality; Tx- Treatment. OS - Overall Survival. CSS – Cancer Specific Survival. DFS – Disease Free Survival. PFS – Progression Free Survival. ADL – Activities of Daily Living. CHF- Congestive Heart Failure. COPD – Chronic Obstructive Pulmonary Disorder. HT- Hypertension. DM- Diabetes Mellitus. CVD – Cardiovascular Disease. TB- Tuberculosis. CCI- Charlson Comorbidity Index. CPD – Chronic Pulmonary Disease. CRF – Chronic Renal Failure. CT - Chemotherapy
